# Proliferating Microglia Exhibit Unique Transcriptional and Functional Alterations in Alzheimer’s Disease

**DOI:** 10.1080/17590914.2025.2506406

**Published:** 2025-05-19

**Authors:** Nàdia Villacampa, Heela Sarlus, Paula Martorell, Khushbu Bhalla, Sergio Castro-Gomez, Ana Vieira-Saecker, Ilya Slutzkin, Kristian Händler, Carmen Venegas, Róisín McManus, Thomas Ulas, Marc Beyer, Eran Segal, Michael T. Heneka

**Affiliations:** a Deutsches Zentrum für Neurodegenerative Erkrankungen (DZNE), Bonn, Germany; b Department of Neurodegenerative Diseases and Geriatric Psychiatry, University of Bonn, Bonn, Germany; c Luxembourg Centre for Systems Biomedicine (LCSB), University of Luxembourg, Belvaux, Luxembourg; d Department of Computer Science and Applied Mathematic, Weizmann Institute of Science, Rehovot, Israel; e Department of Infectious Diseases and Immunology, University of Massachusetts Medical School, Worcester, Massachusetts, USA

**Keywords:** Alzheimer’s disease, inflammasome, microglia, proliferation, transcriptome

## Abstract

Proliferation of microglia represents a physiological process, which is accelerated in several neurodegenerative disorders including Alzheimer disease (AD). The effect of such neurodegeneration-associated microglial proliferation on function and disease progression remains unclear. Here, we show that proliferation results in profound alterations of cellular function by providing evidence that newly proliferated microglia show impaired beta-amyloid clearance in vivo. Through sorting of proliferating microglia of APP/PS1 mice and subsequent transcriptome analysis, we define unique proliferation-associated transcriptomic signatures that change with age and beta-amyloid accumulation and are characterized by enrichment of immune system-related pathways. Of note, we identify the DEAD-Box Helicase 3 X-Linked (DDX3X) as a key molecule to modulate microglia activation and cytokine secretion and it is expressed in the AD brain. Together, these results argue for a novel concept by which phenotypic and functional microglial changes occur longitudinally as a response to accelerated proliferation in a neurodegenerative environment.

## Introduction

Microglia mount an acute immune response against harmful external or internal stimuli including bacterial components or endogenously misfolded or aggregated proteins upon pattern recognition receptor ligation (Venegas & Heneka, 2017). For reasons unknown, the resolution of acute inflammatory responses fails in neurodegenerative disorders and as a result, the innate immune reaction persists (Heneka et al., 2014). The transition from acute to chronic activation diverts microglia from performing beneficial functions and instead leads to the sustained release of proinflammatory mediators, together compromising cognitive functioning (Bachiller et al., 2018; Biswas, 2023; Cisbani & Rivest, 2021; Hickman et al., 2018; Walker et al., 2013). Therefore, finding out the cues leading to chronic inflammation constitute a path in the treatment of chronic neurodegenerative diseases

Under neurodegenerative conditions, chronically activated microglia show enhanced proliferation and cell death together leading to an accelerated turnover of the microglial cell pool (Askew et al., 2017; Doorn et al., 2014; Gómez-Nicola et al., 2013; Hu et al., 2021; Lull & Block, 2010; Ponomarev et al., 2005; Shankaran et al., 2007). Exposed to many new and challenging cues, generated by the persistent inflammation and ongoing neurodegeneration, newly generated microglia unlikely adopt the phenotype of homeostatic microglia that initially populated the brain under healthy conditions. However, the consequences of proliferation for microglial gene expression and function have not been assessed to date.

Recent advances in transcriptomic profiling have identified new subpopulations of microglia and studied their role in aging (Marschallinger et al., 2020; O’Neil et al., 2018) and disease (Chiu et al., 2013; Keren-Shaul et al., 2017; Krasemann et al., 2017). In this work, we describe a novel proliferation-specific genetic signature of microglia in APP/PS1 mice characterized by a strong enrichment of immune system-related pathways at different ages. Interestingly, despite expression of phagocytosis-related genes, proliferating microglia failed to phagocyte Aβ *in vivo*. Using the gene expression data to predict protein–protein interaction (PPI) networks, we identify DEAD-Box Helicase 3 X-Linked (DDX3X) as a potentially important factor expressed in proliferating microglia at 12 months and demonstrate its role in regulating microglia activation through NLRP3 inflammasome. We hypothesize that microglial proliferation in AD generates subpopulations, which contribute to the transition from acute into chronic neuroinflammation and are functionally different from the original microglial cell pool.

## Material and Methods

### Animals

APP/PS1 transgenic mice (The Jackson Laboratory, strain 005864) and ASC^-/-^ mice (Millenium Pharmaceuticals) were backcrossed on C57BL/6 genetic background and aged in-house. APP/PS1, APP/PS1xASC^-/-^ and aged matched wild-type (WT) littermates were housed under standard conditions at 22 °C and a 12 h–12 h light–dark cycle with access to food and water *ad libitum*. Sentinel animals were included for routine health monitoring and screened periodically for common pathogens to ensure a controlled environment. Animal care and handling was performed according to the Declaration of Helsinki and approved by the local ethical committees (LANUV NRW 84-02.04.2017.A226).

### Human Tissue Samples

Post-mortem brain sections from patients with histologically confirmed AD, patients with mild cognitive impairment (MCI), and age-matched controls with no history of other neurodegenerative or inflammatory disease were obtained from Banner Health (USA). AD cases were classified based on established neuropathological criteria, including CERAD neuritic plaque scores and NIA-Reagan (NIA-R) criteria, detailed in the sample table (Table 1). MCI classification was informed by clinical data, such as Mini-Mental State Examination scores, in conjunction with neuropathological assessments. All patients had signed an informed consent by agreeing to donate their brain material for medical research. There were no differences in ages and the post-mortem interval between AD cases and controls. Post-mortem times varied from 2 to 4 h. Patients and controls were 88 ± 10 years old.

**Table 1.** Overview of the human brain tissue samples used in the study.

S.no.	CaseID	Race	Gender	Expired_age	Diagnosis	Braak score	CERAD NP	NIA-R
1	02-24			87	Ctrl	III	Possible AD	Criteria not met
2	98-32	Caucasian	Male	83	Ctrl	III	not AD	Criteria not met
3	04-26	Caucasian	Female	88	Ctrl	III	Possible AD	Criteria not met
4	04-21	Caucasian	Male	89	Ctrl	III	not AD	Criteria not met
5	05-36	Caucasian	Male	82	Ctrl	III	not AD	Criteria not met
6	07-58	Caucasian	Female	88	Ctrl	III	Possible AD	Criteria not met
7	11-15			86	Ctrl	IV	Criteria not met	Criteria not met
8	04-30	Caucasian	Female	78	Ctrl	IV	Possible AD	Criteria not met
9	10-07	Caucasian	Male	89	Ctrl	IV	not AD	Criteria not met
10	06-66	Caucasian	Male	78	Ctrl	III	not AD	Criteria not met
11	97-50	Caucasian	Female	88	MCI	I	not AD	
12	06-13	Caucasian	Female	98	MCI	IV	possible AD	
13	14-24		Male	87	MCI	IV	possible AD	
14	11-93		Male	82	MCI	IV	Criteria not met	
15	12-28		Male	80	MCI	III	Possible AD	
16	04-19	Caucasian	Male	84	MCI	II	not AD	Criteria not met
17	02-01	Caucasian	Female	88	MCI	IV	not AD	Criteria not met
18	06-03	Caucasian	Male	88	MCI	IV	Possible AD	Criteria not met
19	08-59	Caucasian	Male	89	AD	V	Definite AD	High
20	10-79	Caucasian	Female	88	AD	V	Definite AD	High
21	08-01	Caucasian	Male	89	AD	VI	Definite AD	High
22	11-21	Caucasian	Female	88	AD	VI	Definite AD	High
23	07-02	Caucasian	Female	87	AD	V	Definite AD	High
24	10-50	Caucasian	Male	82	AD	V	Definite AD	High
25	08-37	Caucasian	Male	87	AD	VI	Definite AD	High
26	04-24			78	AD	VI	Definite AD	High
27	11-92	Caucasian	Male	83	AD	IV	Definite AD	Intermediate
28	14-43			80	AD	V	Definite AD	Intermediate

**Table 2.** List of research resource identifiers (RRIDs) corresponding to key resources used in the study.

Reagent or resource	Source	Identifier
*Antibodies*		
anti-mouse Ki67	Abcam	Cat# ab15580, RRID:AB_443209
anti-mouse DDX3	Bethyl Laboratories	Cat# A300-474A, RRID:AB_451009
HRP-conjugated goat-anti rabbit	Sigma Aldrich	Cat# 12-348, RRID:AB_390191
anti-Iba1	Wako	Cat# 019-19741, RRID:AB_839504
CD11b-PE	BioLegend	Cat# 101208, RRID:AB_312791
CD45-FITC	eBioscience	Cat# 11-0451-82, RRID:AB_465050
CD16/32 Fc Block	BD Biosciences	Cat# 553141, RRID:AB_394656
Goat anti-rabbit Alexa 594	Invitrogen	Cat# A-11012, RRID:AB_2534079
Goat anti-rabbit Alexa Fluor 647	Thermo Fisher Scientific	Cat# A-21235, RRID:AB_2535804
anti-Caspase-1	AdipoGen	Cat# AG-20B-0042, RRID:AB_2490248
anti-alpha Tubulin	Invitrogen	Cat# 62204, RRID:AB_1965960
anti-beta actin	Cell Signaling	Cat# 4967, RRID:AB_330288
IRDye 800CW Goat anti-Mouse IgG	Li-COR Biosciences	Cat# 926-32210, RRID:AB_621842
IRDye 680RD Goat anti-Rabbit IgG	Li-COR Biosciences	Cat# 926-68071, RRID:AB_10956166
*Software and Algorithms*		
Kallisto	Kallisto	RRID:SCR_016582
DEseq2	DESeq2	RRID:SCR_015687
edgeR	edgeR	RRID:SCR_012802
Metascape	Metascape	RRID:SCR_016620
ImageStudio	Li-COR	RRID:SCR_015795
GraphPad Prism	GraphPad Prism	RRID:SCR_002798

### Perfusion and Tissue Processing

APP/PS1 and WT mice at 4, 6 and 12 months old were anaesthetized by intraperitoneal injection using ketamine and xylazine (100 mg/kg and 10 mg/kg, respectively) and transcardially perfused with cold PBS (30 ml). Brains were dissected out, post-fixed in 4% paraformaldehyde (PFA) for 24 h at 4 °C. After three washes in PBS, the brains were cryopreserved in 30% sucrose for 48 h and subsequently frozen with 2-methylbutane solution (Sigma-Aldrich). Coronal sections of 12 µm-thick were collected on Superfrost slides using a CM3050s Leica cryostat and the slides were stored at −20 °C until their use.

### Immunohistochemistry

Human samples were deparaffinized in Xylene for 10 min and dehydrated in series of alcohol ranging from 99%, 80%, 70%, 50%, and distilled water. Mouse sections were washed in distilled water. Heat induced antigen retrieval was performed on both human and mouse sections using 10 mM sodium citrate buffer (pH = 6.0) by boiling sections in the buffer at 110 °C for 12 min using a pressure cooker. The sections were cooled down for 30 min in the antigen retrieval solution, and for an additional 30 min in distilled water. Tyramide signal amplification (TSA, B40953, Thermo Fisher scientific) was used according to manufacturer’s instructions to amplify the Ki67 or DDX3X staining. The endogenous peroxidase activity was blocked with 3% H_2_O_2_. The sections were washed in PBS, transferred to blocking buffer (BB) containing 5% normal goat serum and 0.3% Triton X-100 in PBS for 30 min and incubated overnight at 4 °C with the rabbit anti-mouse Ki67 (1:500, ab15580, Abcam) or rabbit anti-mouse DDX3 (1:500, A300-474A, Bethyl Laboratories) primary antibodies in BB. The sections were washed three times with PBS for 10 min and incubated with HRP-conjugated goat anti-rabbit (1:4000, 12-348, Sigma) for 2 h. After washing with PBS, the sections were incubated with Tyramide substrate Alexa 488 (1:200) for 30 min at RT. The sections were washed in PBS, blocked with BB, incubated with rabbit anti-Iba1 primary antibody (1:500, 019-19741, Wako) overnight at 4 °C. Sections were washed three times in PBS, incubated with goat anti-rabbit Alexa 594 (1:1000, A-11012, Invitrogen) for 1 h. After subsequent washes in PBS, the amyloid plaques were stained with Methoxy-X04 (863918-78-9, 5 µg/ml, TOCRIS Bioscience) for 15 min, washed in PBS, and mounted using Immu-Mount (9990402, Thermo Fisher Scientific). Nuclei were counterstained with DAPI (4’,6-diamidino-2-phenylindole). Microphotographs were obtained using confocal microscope LSM700 Zeiss.

### Quantification

The Ki67 positive microglia (Ki67 and Iba1 double positive cells) were labelled in the software manually (ZEISS ZEN Imaging software). Ki67 is a nuclear protein therefore overlapping Ki67 staining with the DAPI-stained nuclei along with Iba1 positive cells were labelled as proliferating microglia. The total counts of proliferating microglia were obtained both in human and mouse brain sections. For human samples, counts were obtained independently for the cortex and hippocampus. The total number of proliferating microglia were quantified in the gray and white matter of the entire hippocampus and cortex, and counts were normalized to the respective area to ensure comparability. The total proliferation across conditions was calculated as the average of these normalized values across regions. Each dot in the graph represents this average value obtained from hippocampus and cortex for each subject. For mouse brain sections, proliferating microglia were manually quantified across cortex and hippocampus. 3–4 representative sections were selected per mouse. The obtained counts were then normalized to the respective areas of each region. The normalized values from cortex and hippocampus were combined and averaged across sections to obtain a single value per mouse. These values were used for group-wise comparisons and graphical representation. The area of the sections was measured using Definiens Tissue Software Version 4.3.

### Adult Microglia Isolation for RNA-Sequencing

For obtaining RNA from proliferating microglia the cells underwent fixation as the proliferation maker Ki67 is an intranuclear protein. The method developed by Thomsen ER et al. (2016) with some modifications was used. APP/PS1 mice and WT at 4, 6, and 12 months were intracardially perfused with PBS and the brains were collected in ice cold HBSS. The brains were homogenized using the Adult brain dissociation kit (130 107 667, Biotec Miltenyi) according to the manufacturer’s instructions. The brain homogenates were resuspended in 7 ml HBSS, strained through 40 mM strainer and 3 ml Percoll^®^ were added to obtain a final 30% of Percoll^®^ solution. The samples were subsequently underlayered with 2 ml 70% Percoll^®^ solution using a syringe. The samples were centrifuged at 500 x g with slow acceleration and no brake at 16 °C for 28 min. Microglial cells were recovered from the Percoll^®^ interphase, washed with PBS, centrifuged at 400 x g for 5 min at 4 °C. Thus, the pellet was re-suspended in 4% PFA in PBS and incubated for 15 min on ice. After fixation, the cells were washed with PBS, followed by a wash in staining buffer containing 0.1% RNAse free BSA and 1:400 RNasin plus (N2615, Promega) in PBS, centrifuged at 330 x g for 3 min at 4 °C. The pellet was resuspended in 200 ml staining buffer and frozen at −80 °C until further use.

### FACS Staining and Sorting

The samples were thawed, permeabilized in permeabilization buffer containing 0.1% Triton X100 (T8787, Sigma) and 1:200 Fc-block (rat anti-mouse CD16/32, 553141, BD Biosciences) in staining buffer for 10 min on ice, and centrifuged at 500 x g at 4 °C. The samples were stained in 100 µl primary antibody mix (CD11b-PE, 1:100, 101208, BioLegend; CD45-FITC, 1:100, 11-0451, eBioscience; Ki67, 1:200, ab15580, Abcam) and incubated for 30 min on ice on slow rotation. After centrifugation at 400 x g for 3 min at 4 °C, the samples were stained with secondary anti-rabbit Alexa 647 (1:2000, A-21235, Thermo Fisher scientific) in 200 µl staining buffer for 20 min. The antibody concentrations for Ki67 and secondary antibody were titrated. The samples were washed with staining buffer, centrifuged at 500 x g for 3 min at 4 °C and resuspended in 500 ml staining buffer containing DAPI (10 mg/ml). Unstained samples and fluorescence minus one were used as controls. CD45 and CD11b positive microglia were gated as Ki67 positive and Ki67 negative cells and DAPI staining confirmed that cells containing nuclei were sorted. Using BD FACSAria II 400 proliferating or non-proliferating microglia respectively were collected in 1.5 ml RNase free containing 25 ml PKD buffer supplemented with 1:16 proteinase K (RNeasy FFPE kit, 73504, Qiagen) and ERCC spike in controls (4456739, Thermo Fisher scientific), snap frozen on dry ice and stored at −80 °C until further processing.

### Assessment of Aβ Phagocytosis by FACS

APP/PS1 and APP/PS1xASC^-/-^ mice were injected with 10 mg/kg of Methoxy-X04 (863918-78-9, TOCRIS Bioscience) in 50% DMSO and 50% NaCl (0.9% at pH = 12) and 3 h later they were analyzed as previously described (Tejera and Heneka, 2019). The microglia population was isolated from mice and stained with primary antibody mix (CD11b-PE, 1:100, 101208, BioLegend; CD45-FITC, 1:100, 11-0451, eBioscience; Ki67, 1:200, ab15580, Abcam). After gating into CD11b^+^/CD45^+^; methoxy-X04^+^ (phagocytic) and Ki67^+^ (proliferating) microglia were sorted and the numbers were determined by using BD FACS Aria II.

### RNA Isolation and cDNA Preparation

After obtaining sorted microglia, RNA isolation and cDNA synthesis protocols were adapted from Thomsen et al. (2016). Briefly, to lyse the sorted cells, the samples were thawed at RT, 75 µl of Proteinase K digestion buffer (PKD buffer) was added and incubated for 1 h at 56 °C at 300 rpm for reverse cross-linking. The PKD buffer was supplied as a component of the miRNeasy FFPE Kit. Samples were then centrifuged at 20.000 x g for 20 min. For RNA extraction, the miRNeasy FFPE Kit (217504, Qiagen) was used following manufacturer’s instructions to obtain 14 µl of RNA. The samples were incubated for 3 min at 72 °C in a lysis mix, and the reverse-transcriptase mix was added and the reaction was performed at 42 °C for 90 min and 70 °C for 15 min. Then, the pre-amplification mix was added and the reaction was performed for 24 cycles (98 °C for 20 s, 67 °C for 20 s and 72 °C for 6 min). The cDNA was purified using Ampure XP Beads (A63881, Beckmann) at 1:1 ratio and eluted in 15 µl of nuclease-free water. Samples were quantified using a High Sensitivity D5000 DNA assay (Agilent, 5067-5592) on an Agilent 2200 Tapestation.

### Library Construction and Sequencing

Purified full-length cDNA was processed to NGS libraries using the Nextera XT DNA library preparation and the Nextera XT Index kits (Illumina). In short, cDNA was diluted to 200 pg/µl, 100 pg were tagmented using 0.5 µl Amplicon Tagment Mix (ATM) at 55 °C for 8 min. Tagmentation was terminated by addition of 0.5 µl Neutralize Tagment Buffer (NT buffer). 1 µl primer mix and 1.5 ml NPM were added and tagmented cDNA was PCR amplified using the following protocol: 72 °C for 5 min, 98 °C for 30 s, 16 cycles of 98 °C for 10 s, 63 °C for 30 s, 72 °C for 1 min, then 72 °C for 5 min. The finished NGS libraries were purified using Ampure XP Beads (A63881, Beckmann) at a 1:1 ratio and eluted in 15 µl of nuclease-free water before quantification and QC using a High Sensitivity D5000 DNA assay (Agilent, 5067-5592) on an Agilent 2200 Tapestation. Libraries were sequenced on a Nextseq500 system using high output chemistry V1 (75 bp SR, or 75 bp & 75 bp PE, Illumina). Base calling from base call files and file conversion to fastq files were achieved by Illumina standard pipeline scripts (bcl2fastq2 v.2.20). Data was demultiplexed, and only read 1 of paired-end runs used for subsequent analysis. After merging, samples were aligned to the mouse GRCm38 reference genome using Kallisto v0.43.1 (Bray et al., 2016) and count matrixes calculated using the DESeq2 package in R (Love et al., 2014) with subsequent normalization and batch-correction using DEseq2 SVA10.

### RNA-Seq Analysis

Differential expression analysis was conducted using a GLM framework with genotype, time point and proliferation state as factors in edgeR, with FDR (False Discovery Rate) adjustment at a significance level of 0.05 to account for multiple comparisons. For comparisons of transcriptome changes in proliferating microglia with published datasets; we selected the following published RNA-seq data sets of microglia: DAM versus homeostatic microglia (Keren-Shaul et al., 2017); lipid-droplet^low^ vs lipid-droplet^high^ accumulating microglia (Marschallinger et al., 2020) and MGnD versus homeostatic microglia (Krasemann et al., 2017). Gene ontology analysis was performed using Metascape (Zhou et al., 2019). Interactions among genes were analyzed using String online and Pathway Commons.

### Cell Culture

Primary mouse microglia cell cultures were prepared as previously described from newborn wild-type pups (Venegas & Heneka, 2017). Briefly, brains from neonatal (P0–P3) mice were stripped of the meninges and dissociated using mechanical shearing and trypsin. Cells of two brains were plated on PLL-coated T75 culture flasks and cultivated in DMEM supplemented with 10% heat-inactivated fetal bovine serum and 1% penicillin/streptomycin. On the next day, cells were washed three times with PBS to remove cellular debris and cultured with DMEM supplemented with 10% FBS, 1% P/S and 10% L929 conditioned medium as a source of growth factors. After approximately 7-10 days, loosely attached mature microglia were shaken off the astrocytic monolayer with a repetition of the harvesting procedure at every 2–3 days for up to three times. Cells were seeded in DMEM, 1% P/S supplemented with N-2 and allowed to adhere overnight. For NLRP3 stimulation assays, microglia were primed with 100 ng/ml of ultrapure LPS (E. coli 0111:B4, Invivogen) for 3 h and further activated with 2.5 μM of nigericin (Invitrogen) for 30 min. The DDX3X inhibitor (RK-33, S8246, Selleckchem) was added in the culture medium 30 min before the priming step and maintained throughout the treatment when tested.

### Cell Viability/Cytotoxicity Assays and Measurement of IL-1β

Cell viability/cytotoxicity of the inhibitor RK-33 was measured using two different assays: XTT assay and LDH release. The metabolic activity of primary microglia was quantified using the XTT Cell Viability Kit (9095, Cell Signaling Technology). Primary microglia were seeded at fifty thousand cells per well into 96-well plates and treated with 100 μl medium per well. After treatment with the various stimuli, supernatants were collected and frozen at −80 °C or used directly. The XTT Reagent and Electron Coupling Solution were added to the cells with fresh cell culture medium and incubated for 4 hours, according to the manufacturer’s protocol. A quantity of 50 μl of collected cell supernatants was used for the cytotoxicity assay (LDH cytotoxicity detection kit, 11644793001, Sigma-Aldrich) according to the manufacturer’s protocol. Inhibition of IL-1β release was determined by measuring the IL-1β secretion in the supernatants using the mouse IL-1 beta/IL-1F2 DuoSet ELISA kit (DY401, R&D Systems). The concentration of cytokine was quantified using the relevant standard curves. Absorbances were measured at 450 nm using on Infinite 200 PRO plate reader (Tecan).

### Adult Microglia Isolation for Immunoblot

Brains were collected from APP/PS1 and WT at 12 months. After removing the cerebellum and olfactory bulbs, each brain was dissociated using Adult Brain Dissociation Kit (130-107-677, Miltenyi Biotec, Bergisch Gladbach, Germany). From the resulting brain homogenates, microglial cells were enriched using a magnetic-bead-coupled anti-CD11b (130-126-725, Miltenyi Biotec, Bergisch Gladbach, Germany) according to the manufacturer’s protocol. After column elution, isolated cells were washed in 1 ml of DPBS, incubated in 2x RIPA buffer (50 mM Tris-HCl, 150 mM NaCl, 2% NP40, 1% sodium dodecylsulfate, 0.2% SDS) and 1x protease/phosphatase inhibitor (Cell Signaling) for 30 min, centrifuged at 15000 x g for 10 min and stored at −80 °C.

### Immunoblot Analysis

For standard immunoblot analysis, 20 μg of protein samples were supplemented with 1× NuPAGE sample buffer, heated for 5 min at 95 °C and loaded on 4–12% gels (WG1401BOX, NuPAGE). After transfer of proteins to nitrocellulose membranes, membranes were blocked with 5% BSA in TBS-Tween followed by overnight incubation the following primary antibodies: rabbit anti-DDX3 (A300-474A, Bethyl Laboratories); mouse anti-Caspase-1 (p20), mAb (Casper-1) (AG-20B-0042, AdipoGen); mouse anti-alpha Tubulin (62204, Invitrogen), rabbit anti-beta actin (4967, Cell Signaling). Visualization of proteins was achieved by using fluorescent-tagged secondary antibodies IRDye 800CW Goat anti-Mouse IgG (926-32210, Li-COR) and IRDye 680RD Goat anti-Rabbit IgG (926-68071,Li-COR). Imaging was performed by using a LI-COR ODYSSEY CLx. Data were analyzed with ImageStudio software version 5.2.5 (LI-COR). All controls were run as loading controls on the same gel.

### Statistics

All statistical comparisons were performed using GraphPad Prism 6 for Mac OS or R. Unpaired two-tailed Student’s t-test, one-way ANOVA, or two-way ANOVA with appropriate post-hoc tests were used as applicable. Statistical details are given in the respective figure legends.

## Results

### Microglial Dynamics in a Chronic Neurodegenerative Environment

**Figure 1.**
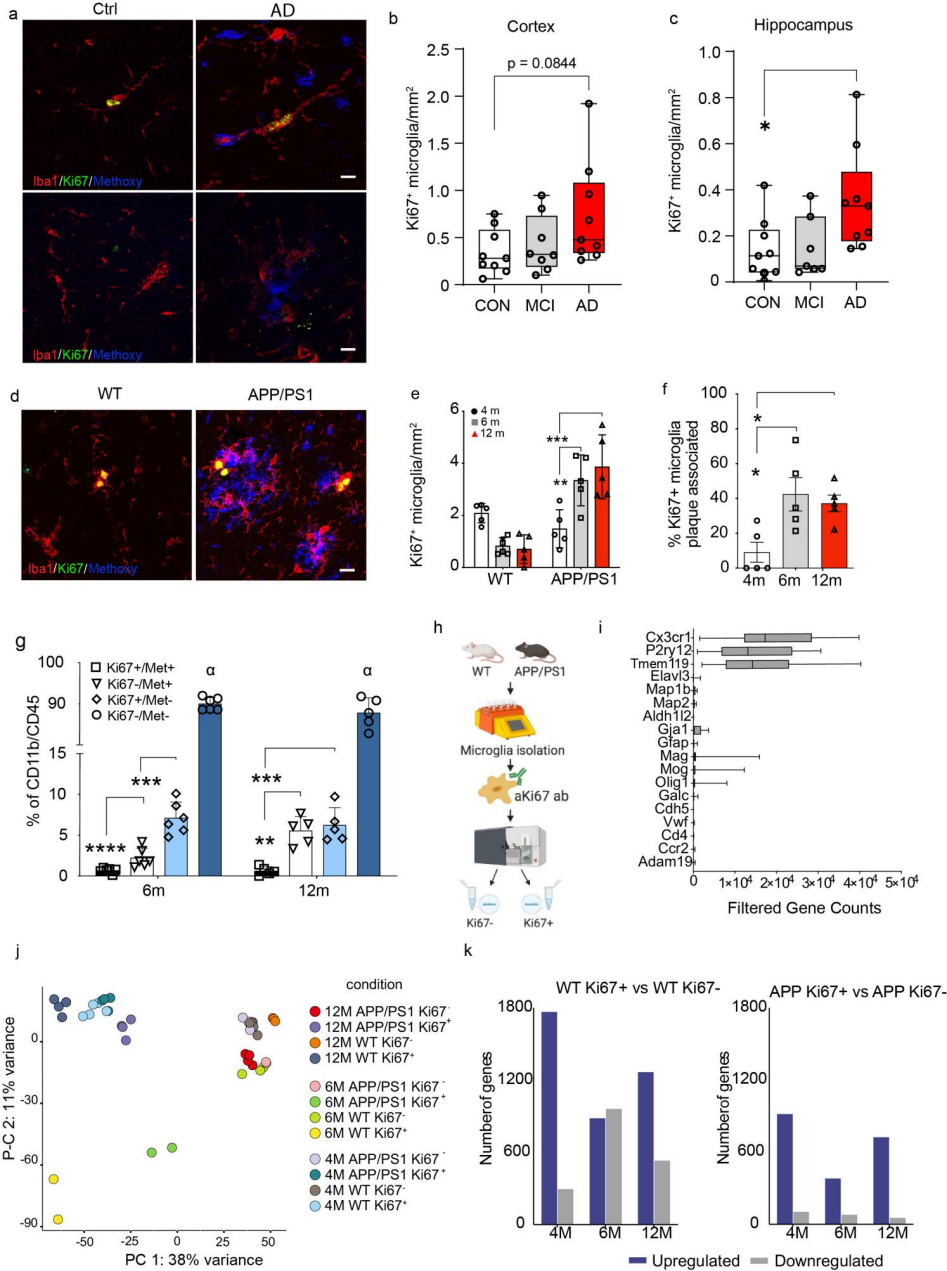
Dynamics and phenotype of microglial proliferation in Alzheimer’s disease. **A)** Proliferating microglia stained with Ki67 (green), beta-amyloid plaque (blue) and microglia stained with Iba1 (red) in the cortex (upper panel) and hippocampus (lower panel) of Ctrl and AD brains (scale bar = 10µm). **B)** Quantification of proliferating microglia in cortex in control human cases (Ctrl) (n = 9), mild cognitive impairment (MCI) (n = 8) and Alzheimer’s (AD) (n = 9). One-way ANOVA with Tukey’s multiple comparisons test. **C)** Quantification of proliferating microglia in hippocampus in control human cases (Ctrl) (n = 9), mild cognitive impairment (MCI) (n = 7) and Alzheimer’s (AD) (n = 9). One-way ANOVA with Tukey’s multiple comparisons test, * indicates p < 0.05 in AD compared to Ctrl. **D)** Proliferating microglia stained with Ki67 (green), beta-amyloid plaque stained with Methoxy (blue) and microglia stained with Iba1 (red) in WT and APP/PS1 mice at 12 months. **E)** Quantification of proliferating microglia in WT and APP/PS1 mice at 4 (n = 5/genotype), 6 (n = 5/genotype) and 12 months (n = 5/genotype). Two-way ANOVA with Holm-Sidak’s multiple comparisons test, ** indicates p < 0.01 compared to 4 months, *** indicates p < 0.001 compared to 6 months. **F)** Quantification proliferating microglia associated to an amyloid plaque in APP/PS1 mice at 4 (n = 5), 6 (n = 5) and 12 months (n = 5) expressed as % of total proliferating microglia. One-way ANOVA with Sidak’s multiple comparisons test * indicates p < 0.05 compared to 4 months. **G)** Quantification of amyloid content (Met^+^) and proliferation (Ki67^+^) in CD11b^+^/CD45^+^ microglia in APP/PS1 mice at 6 (n = 6) and 12 months (n = 5). Double positive microglia were almost absent. Two-way ANOVA with Tukey’s post hoc test ** indicates p = 0.0010, *** indicates p = 0.0002, **** indicates p < 0.001. **H)** Schematic figure representing the experimental design to obtain proliferating microglia from WT and APP/PS1 mice at 4, 6 and 12 m for bulk RNA-seq. **I)** Filtered gene counts of microglia specific genes demonstrating purity of the cells obtained. **J)** PCA plot showing unsupervised clustering of groups by both proliferation state (PC1) and age (PC2). **K)** Bar plots of DEGs upregulated (blue) and downregulated (grey) genes between proliferating and non-proliferating cells at 4, 6 and 12 months for WT (left) and APP/PS1 (right) mice.

We first assessed the dynamics of microglial proliferation in APP/PS1 mice and brains from AD cases. In AD brains, a mild increase of microglial proliferation was found compared to healthy age-matched controls in the cortex and hippocampus (Figure 1a–c). In both, wild-type (WT) and APP/PS1 a similar number of proliferating Ki67^+^ microglia were found at 4 months, prior to any Aβ deposition. Subsequently, microglial proliferation decreases in WT animals, but increases progressively in APP/PS1 mice (Figure 1d and e). Spatial distribution analysis revealed that around 42% of proliferating microglia were located at sites of Ab deposition at 6 and 12 months (Figure 1f). Compromised microglial clearance has been suggested to represent a key mechanism for the development of sporadic AD (Mawuenyega et al., 2010). To test whether proliferating microglia show functional impairment and in particular a reduced capacity for Aβ clearance, we assessed microglial Aβ phagocytosis *in vivo* as described previously using the methoxy-X04 assay (Heneka et al., 2013). These experiments revealed that Ki67^+^ microglia incorporated significantly less Aβ as compared to non-proliferating Ki67^-^ microglia, pointing to a functional clearance deficit in microglia that had undergone proliferation (Figure 1g).

### Proliferation Induced Changes of the Microglial Transcriptome

To test if a chronic neurodegenerative environment will change the microglial phenotype through proliferation, we sorted proliferating CD11b^+^/CD45^+^/Ki67^+^ microglia and non-proliferating CD11b^+^/CD45^+^/Ki67^-^ microglia from WT and APP/PS1 male mice at 4, 6 and 12 months. Up to 400 cells were collected in 25 µl PKD, whether proliferating or non-proliferating. As cells required fixation after Ki67 nuclear stain, we used the FRISCR method and subsequently analyzed by RNA-sequencing (Figure 1h). The purity of samples was determined by visualizing a selected set of genes showing that homeostatic microglial markers including *Cx3Cr1*, *P2ry12*, and *Tmem119* were highly expressed, whereas genes characterizing neuronal (*Elavl3*, *Map1b* and *Map2*), astroglial (*Aldh1l2*, *Gfa1* and *Gfap*), oligodendroglial (*Mog*, *Mag* and *Olig1*), endothelial (*Galc*, *Cdh5* and *Vwf*) or peripheral immune cell populations (*Cd4*, *Ccr2* and *Adam19*) were almost undetectable (Figure 1i, Supplementary Table 1). To identify the main sources of variability in gene expression between our samples, we performed a principal component analysis (PCA). Here, proliferation as detected by Ki67 immunopositivity, was clearly associated with the first principal component. At all-time points, proliferating microglia formed a separate cluster compared to non-proliferating microglia in WT and APP/PS1 mice (Figure 1j). Remarkably, at 12 months proliferating microglia in APP/PS1 mice form a clear cluster, separated from non-proliferating microglia in APP/PS1 mice but also from proliferating microglia in WT animals (Figure 1j). Next, we performed differential gene expression analysis on the acquired gene counts using edgeR. We applied a generalized linear model (GLM) with genotype, time point and proliferation state as factors. A comparison of the FDR corrected p-values between groups revealed that the proliferation state had the strongest effect (Supplementary Figure 1), supporting the prior PCA analysis. To examine changes in proliferation-associated genes across time, we queried our GLM to identify those genes that were differentially regulated in proliferating vs non-proliferating microglia in WT and APP/PS1 mice at each time point. This analysis revealed that a considerable number of differentially expressed genes (DEGs) could be detected at different time points (Figure 1k). To explore changes in proliferation-associated DEGs between groups; we examined the differences between Ki67^+^ and Ki67^-^ cells in WT and APP/PS1 mice over time (Supplementary Table 2). A substantial number of genes upregulated in Ki67+ APP/PS1 microglia (n = 378 and n = 724 at 6 and 12 months, respectively) overlapped between APP/PS1 and WT mice at 6 (n = 300) and 12 months (n = 438) with a large proportion being shared between both time-points (n = 152) (Supplementary Figure 1). However, proliferating microglia of APP/PS1 mice expressed a specific gene signature over the experimental trajectory with 41 and 198 non-overlapping genes at 6 and 12 months, respectively (Supplementary Figure 1). As expected from the overall analysis of downregulated genes in Ki67^+^ microglia, there was very little overlap between APP/PS1 and WT microglia (Supplementary Figure 1). Taken together, these data support that Ki67+ microglia are clearly distinct from non-proliferating microglia and that amyloid deposition induces an additional signature in Ki67^+^ microglia.

### Disease-Associated Gene Signature of Proliferating Microglia

**Figure 2.**
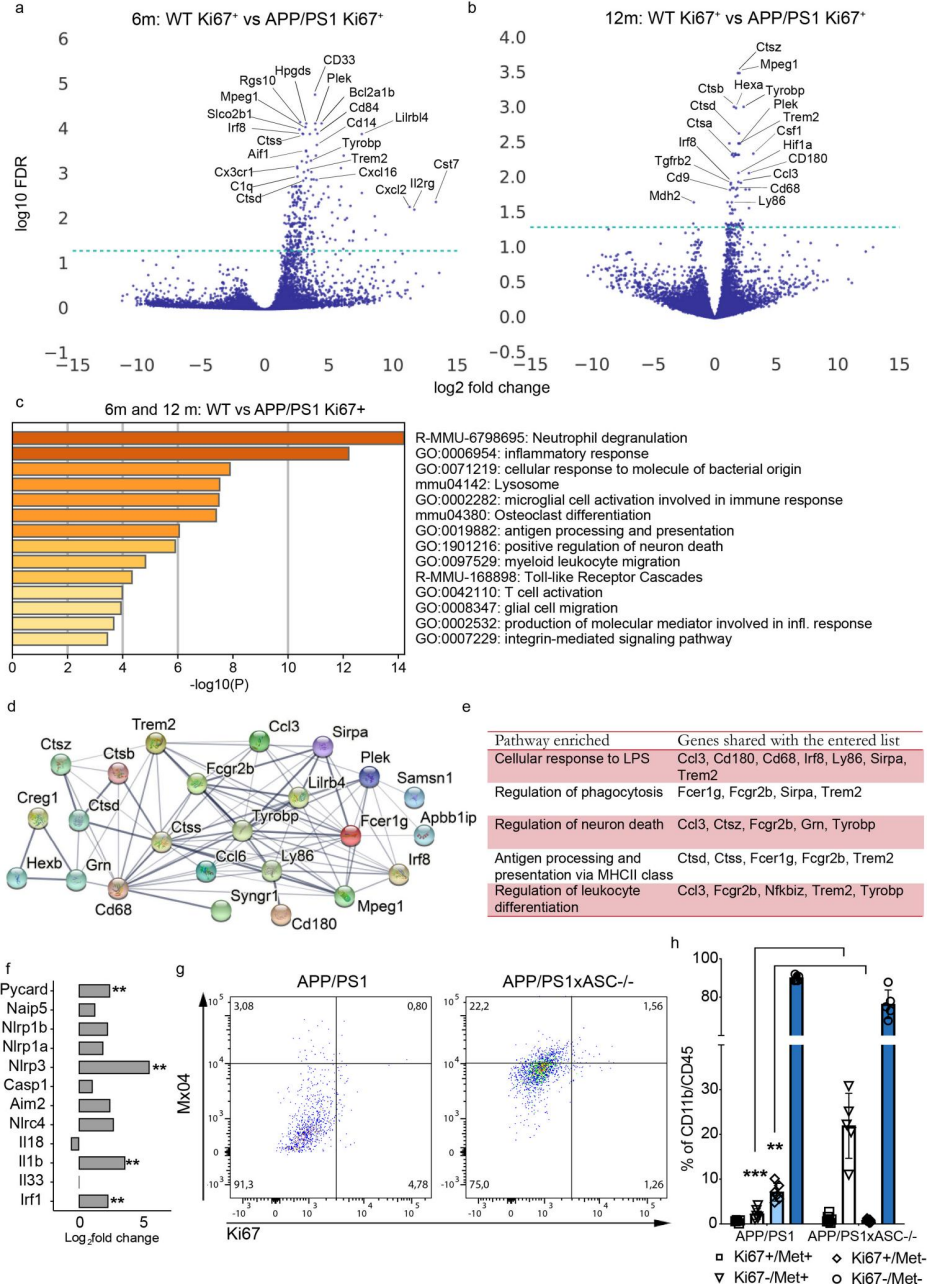
Effects of disease in the transcriptome of microglia. **A,B)** Volcano plots for disease associated DEGs for proliferating cells at 6 m and 12 m in WT vs APP/PS1. **C)** Gene ontology analysis of 35 upregulated genes shared between APP vs WT Ki67^+^ microglia at 6 m and 12 m. **D)** Protein-protein interaction network form STRING online analysis website from the DEGs found at 6 m and 12 m between APP vs WT Ki67^+^ microglia. Network nodes represent proteins and edges represent protein-protein associations. Empty nodes represent proteins of unknown 3D structure, while filled represent proteins with predicted or known 3D structure (PPI enrichment value <1.0e-16). **E)** Pathway enrichment analysis of the 35 upregulated genes shared at 6 m and 12 m. **F)** Graph representing inflammasome-related genes in proliferating microglia of APP/PS1 mice at 6 m when compared to WT. ** indicates p < 0.01. **G)** Representative scatter plots of mice analyzed for microglial amyloid content after intraperitoneal injection of methoxy-X04 (Mx04) **H)** Quantification of amyloid content (Met^+^) and proliferation (Ki67^+^) in CD11b^+^/CD45^+^ microglia in APP/PS1 and APP/PS1xASC^-/-^ mice at 6 months. Two-way ANOVA with Tukey’s multiple comparisons test, ** indicates p < 0.01 compared to Ki67^+^ microglia in the APP/PS1xASC^-/-^, *** indicates p < 0.001 compared to Mx04^+^ microglia in the APP/PS1xASC^-/-^ mice. (n = 6 APP/PS1, n = 6 APP/PS1xASC^-/-^ mice).

To further examine whether proliferating microglia are characterized by a disease-associated gene signature, we queried our GLM to identify differentially expressed genes in proliferating microglia in WT vs proliferating microglia in APP/PS1 and likewise for non-proliferating microglia at all time-points (Supplementary Table 1). While there aren’t any significant differences at 4 months, proliferating microglia start to display DEGs at 6 and 12 months in APP/PS1 animals. Briefly, a total of 255 DEGs were upregulated and 1 DEG was downregulated at 6 months while a total of 61 DEGs were upregulated and 2 DEGs were downregulated at 12 months (Supplementary Figure 1). DEGs in proliferating microglia in APP/PS1 mice presented little over-lap with DEGs in non-proliferating microglia (Supplementary Figure 1). When focusing on the upregulated DEGs in proliferating microglia of APP/PS1 mice, 32 genes are expressed at both 6 and 12 months, while 223 and 29 genes are uniquely expressed at 6 and 12 months respectively (Supplementary Figure 1). At 6 months, proliferating microglia upregulate several well-described microglia genes including Irf8, Cx3cr1, Csf1r, Aif1 and Siglech. In this microglial subpopulation the Cd33 gene showed the highest significance in the group of upregulated genes (Figure 2a). Trem2, which acts downstream of CD33 (Griciuc et al., 2019), was concomitantly upregulated. Of note, both genes are associated with increased risk of AD (Guerreiro et al., 2013; Hollingworth et al., 2011). At 12 months, the upregulated gene showing the lowest p-value was *Mpeg1*, a perforin-like protein bound to the phagolysosome membrane (Pang et al., 2019). In addition, *Trem2* and *Tyrobp* remained upregulated at 12 months, indicating that the TREM2 signaling pathway stays activated. Other DEGs also included several cathepsins (*Ctsz*, *Ctsb*, *Ctsd* and *Ctsa*), the microglial sensome genes *Ly86*, *Tgfrb2* and *Cd180* (Hickman et al., 2013) and previously as DAM genes denoted genes such as *Cd68*, *Hif1a* and *Cd9* (Figure 2b). Functional characterization using GO-terms or KEGG pathways revealed strong involvement of immune pathways along the progression of the disease as neutrophil degranulation, inflammatory response and leukocyte migration (data not shown). Interestingly, in non-proliferating microglia, DEGs were not detectable before 12 months of age (Supplementary Figure 1). This may indicate an aging effect of non-proliferating microglia that causes a delayed transcriptional change, after sustained challenge by a neurodegenerative environment. In these microglia, *Tecpr1*, involved in autophagosome formation, was the most upregulated gene. Several other DEGs related to palmitoylation (*Zdhhc4*), hydrolase activity (*Haghl*), fatty-acid binding (*Fabp5*), cellular movement (*Myo1e*), monocyte chemotaxis (*Cxcl14*) and secretase activity (*Psen2*) were also upregulated in non-proliferating microglia of APP/PS1 mice at 12 months (Supplementary Figure 2). Functional annotation of these changes in non-proliferating microglia revealed alterations in metabolic pathways instead of immune processes (Supplementary Figure 2).

### Microglia Change Their Transcriptome and Function over Time

We assessed common features of proliferating microglia in a neurodegenerative environment at 6 and 12 months. In total 32 DEGs overlapped between 6 and 12 months in this microglial subpopulation (Supplementary Figure 1). Enriched pathways primarily related to immune system functions including neutrophil degranulation, inflammatory response, cellular response to molecule of bacterial origin and the lysosome (Figure 2c). Using the search tool for retrieval of interacting genes (STRING), we studied the known interactions among these genes (Figure 2d). In particular, DEGs were related to cellular response to lipopolysaccharide, regulation of phagocytosis, regulation of neuron death, antigen processing and presentation by MHC class II and regulation of leukocyte differentiation (Figure 2e).

Proliferating microglia in the APP/PS1 mice at 6 months express several inflammasome related genes, such as *Pycard*, *Nlrp3*, *Il1b* and *Irf1* (Figure 2f). As suggested in a previous work (Heneka et al., 2013), inflammasome activation may prevent microglial-mediated phagocytosis of Aβ. To test the role of inflammasome in phagocytosis of Ab in vivo, we used ASC knockout mice backcrossed to the APP/PS1 line (APP/ASC^-/-^). APP/PS1 and APP/ASC^-/-^ mice received an i.p. injection of methoxy-X04 as previously described (Heneka et al., 2013). Microglia were subsequently sorted and analysed for the incorporation of Ab and Ki67 expression. After CD11b^+^/CD45^+^ gating, only 0.62% in the APP/PS1 and 1,28% in the APP/PS1/ASC^-/-^ microglia were Ki67^+^/Methoxy^+^, indicating a strong impairment of phagocytic Aβ clearance by proliferating microglia in APP/PS1 (Figure 2g and h). Interestingly, the total amount of proliferating microglia was significantly lower in APP/PS1xASC^-/-^ mice. In addition, non-proliferating microglia of APP/PS1/ASC^-/-^ showed a much higher Aβ clearance when compared to the APP/PS1 mice (Figure 2g and h), indicating that inflammasome signalling through ASC compromises microglial clearance.

**Figure 3.**
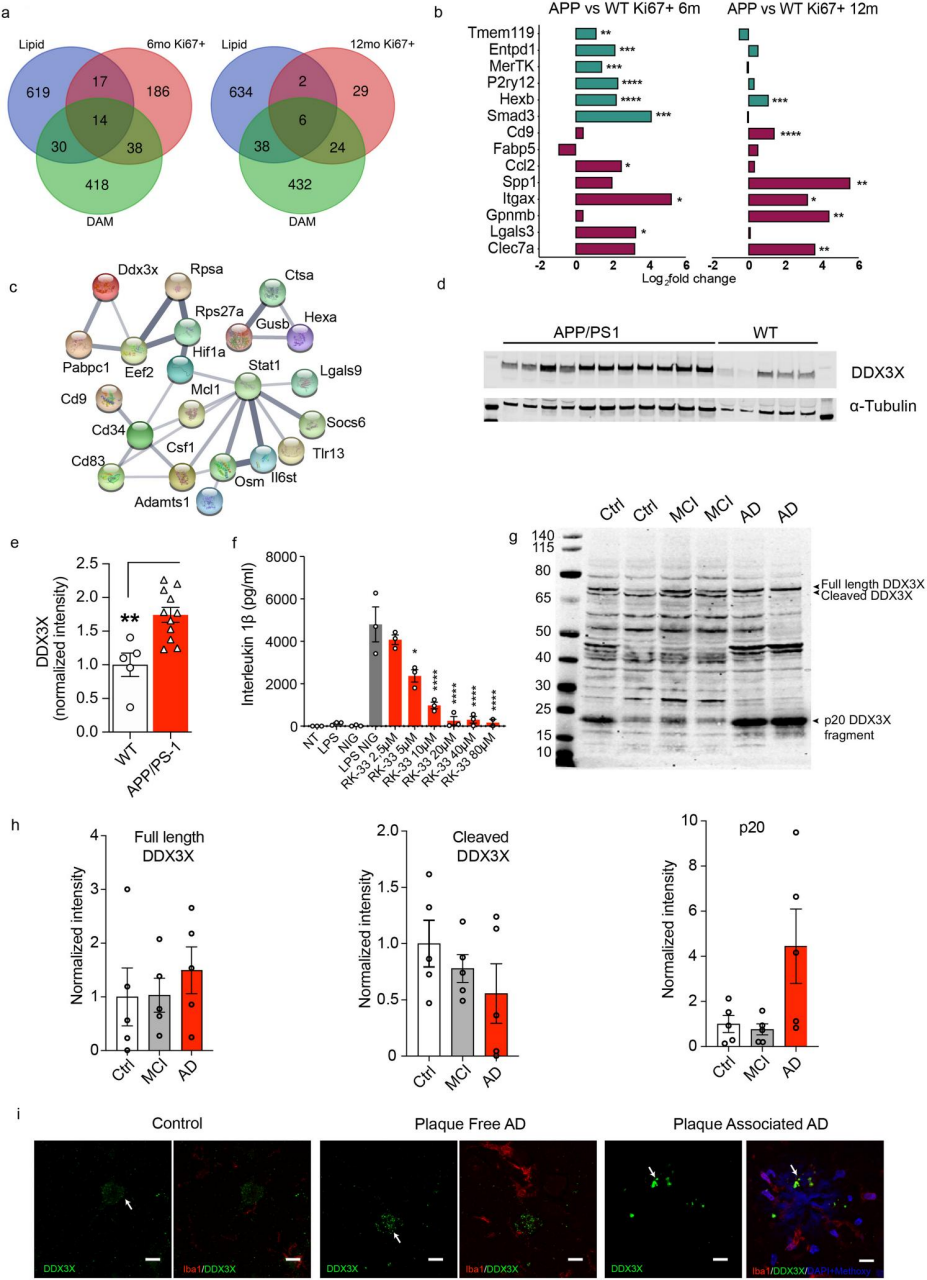
DDX3X is expressed on microglia in Alzheimer’s disease. **A)** Venn diagrams representing overlap with lipid^high^ microglia of 18 m mice (lipid, purple) and the disease associated microglia (DAM, green) with APP vs WT Ki67^+^ microglia at 6 m (6 m Ki67^+^, left, red) and APP vs WT Ki67^+^ microglia at 12 m (12 m Ki67^+^, right, red). **B)** Expression of several genes associated with tolerogenic (green) and neurodegenerative (red) microglia in proliferating microglia of APP/PS1 compared to WT at 6 m (left) and 12 m (right). * indicates p < 0.05, ** indicates p < 0.01, *** indicates p < 0.001, **** indicates p < 0.0001. **C)** Protein-protein interaction network form STRING online analysis from DEGs found uniquely at 12 m. Network nodes represent proteins and edges represent protein-protein associations. Empty nodes represent proteins of unknown 3D structure, while filled represent proteins with predicted or known 3D structure (PPI enrichment value = 0.000134). **D)** Blots of cell lysates of isolated adult WT and APP/PS1 microglia at 12 m stained for DDX3X and alpha-tubulin, as a loading control. **E)** Quantification of DDX3X levels in isolated adult microglia, normalized to alpha-tubulin. WT (n = 5), APP/PS1 (n = 11), Student’s t-test, ** indicates p < 0.01 compared to the corresponding time point in the WT. **F)** Release of IL-1β and cytotoxicity in primary WT microglia cells after NLRP3 inflammasome activation and treatment with increasing concentrations of DDX3X inhibitor (RK-33). **G)** Blots of human brain homogenates from control (Ctrl), mild cognitive impairment (MCI) and Alzheimer’s disease (AD) stained for DDX3X. **H)** Quantification of the three DDX3X fragments in human brain homogenates from control (Ctrl) (n = 5), mild cognitive impairment (MCI) (n = 5) and Alzheimer’s disease (AD) (n = 5), normalized to alpha-tubulin. **I)** Representative microphotographs of human brain from controls (Ctrl) and AD patients (AD) showing DDX3X (green) staining in the neuronal soma in plaque-free regions and in microglia (anti-Iba1, red) in plaque regions, stained with Methoxy (blue).

In order to study the transcriptional changes longitudinally, Ki67^+^ microglia from APP/PS1 mice at 6 and 12 months were compared to existing data sets. At 6 months, 52 of 255 upregulated DEGs (20%) were shared with the DAM signature, whereas at 12 months, 30 out of 61 (49%) overlapped with that signature (Keren-Shaul et al., 2017) (Figure 3, Supplementary Table 2). In contrast, only little overlap was found with the genetic signature of lipid-droplet accumulating microglia (LDAM) (Marschallinger et al., 2020) at both ages (Figure 3a). When comparing the genetic signature identified in the present work with subpopulations defined by Krasemann and colleagues as “tolerogenic microglia” and “neurodegenerative microglia” (Krasemann et al., 2017), we observed that along with aging and amyloid deposition, proliferating microglia develop a neurodegeneration-associated phenotype while losing their “tolerogenic” phenotype (Figure 3b).

When we focused onto the 6-month time-point to better understand the functional association of the interaction network obtained with the STRING analysis, we found enrichment in the pathways related to G-protein beta-gamma signalling, substrate-dependent cell migration, leukocyte apoptotic process, toll-like receptor cascades and regulation of lipid biosynthetic process at 6 months (Supplementary Figure 3). Using this approach, we next identified the functional network within the 29 genes upregulated at 12 but not 6 months that might be involved in the transition from acute to chronic inflammation (Figure 3c). In fact, the involved pathways were interleukin-6 family signalling, interleukin-4 and -13 signalling, uptake and function of diphtheria toxin, and two pathways related to transforming growth factor (TGF)-β production (Figure 3c). From the reconstructed network, we selected DDX3X as a potential candidate for modulating microglia function, since DDX3X is involved in NLRP3 inflammasome regulation (Kesavardhana et al., 2021; Kienes et al., 2021; Samir et al., 2019) and NLRP3 inflammasome activation in microglia contributes to pathology in APP/PS1 and tau22 mice (Heneka et al., 2013; Ising et al., 2019). *In vivo*, DDX3X is significantly increased in isolated microglia from brains of APP/PS1 mice when compared to WT at 12 months (Figure 3d). *In vitro*, DDX3X inhibition efficiently reduces IL-1β production and rescues cell death after NLRP3 activation (Figure 3f, Supplementary Figure 4), concomitant with a reduction of cleaved-caspase-1 in both supernatants and cell lysates (Supplementary Figure 4). Importantly, we detected three DDX3X fragments in human brain samples: the previously described ∼70 kDa active fragment and the ∼65 kDa inactive cleaved fragment (Sun et al., 2008); and a 20 kDa fragment, which might contain the helicase domain (Rodamilans & Montoya, 2007) (Figure 3g). In the AD brain, a trend is observed toward higher levels of active fragment and 20 kDa fragment together with decreases levels of the inactive DDX3X fragment (Figure 3h). In control brain and plaque-free regions in the AD brains, DDX3X is found in the neuronal soma; however, in plaque-loaded regions, DDX3X is found in microglia in close relationship to amyloid plaques (Figure 3i).

## Discussion

Enhanced proliferation represents a key feature of the microglial response to acute injuries and chronic neurodegeneration. Using RNA-sequencing, we describe for the first time the unique transcriptome of proliferating microglia derived from 6 and 12 months-old APP/PS1 mice. We show that proliferating microglia already exhibit transcriptional changes related to AD at 6 months, suggesting that newly generated microglia adopt a distinct transcriptome upon early cerebral amyloidosis. The identified pathways mainly relate to immune functions indicating a role of proliferating microglia in sustaining the immunological response upon exposure to accumulating amyloid deposition. Proliferating microglia showed an upregulation of several “microglial sensome” genes (Hickman et al., 2013) and also the *Trem2* co-adaptor molecule *Tyrobp*, which has been linked to enhanced phagocytic activity (Gaikwad et al., 2009). Other phagocytosis-related genes such as *Cd68* (Schafer & Stevens, 2015) and lysosomal genes including several cathepsins (Lowry & Klegeris, 2018) were also upregulated. Such upregulation of phagocytosis-related pathways was striking as proliferating microglia were strongly impaired in Abeta phagocytosis *in vivo*. Thus, such transcriptome changes may have to be interpreted as an attempt to overcome the functional impairment through an upregulated gene transcription. In 5xFAD mice, a neuroprotective microglial subtype denoted as DAMs, with high phagocytic capacity often associated with Aβ deposition and whose full activation depends on TREM2 signaling, has been described (Keren-Shaul et al., 2017). While DAM are associated with effective phagocytosis, proliferating cells might get arrested in an intermediate state and do not achieve an efficient phagocytic phenotype. In this work, we provide a list of 31 DEGs found in proliferating microglia at 12 months that do not overlap with the DAM signature, which may account for this functional phenotype. Whether some of these genes may serve as future targets to boost microglial clearance in the context of neurodegenerative diseases will require further studies.

In this work, we provide 29 genes upregulated uniquely at 12 m that might represent key players in the acquisition of a chronically activated microglial phenotype. Five of these genes are also found in the second phase of DAM activation (*Csf1*, *Ctsa*, *Hif1a*, *Cd9*, *Gusb*). Interestingly, 12 of these 29 DEGs have not been described previously in the context of either AD or in microglia such as *Fam102b*, *Pabpc1*, *Sdf4*, *Rps27a*, *Ptpra* and *Lars2*. Furthermore, we found upregulation of *Socs6* and *Tlr13*, which have been reported in microglia but not described in the context of AD before. Mice lacking RNA-sensing endosomal *Tlr13* were susceptible to encephalitis and *Tlr13*^-/-^ microglia showed decreased cytokine production *in vitro* (Famà et al., 2020). *Socs6* was upregulated after TGF-β1 treatment in primary microglia (Zhou et al., 2015). In addition, proliferating microglia at 12 m upregulated the stem cell marker *Cd34*, which identifies a subset of proliferating microglia associated with degenerating neurons in ALS (Kovacs et al., 2019) and which is upregulated in early-activated microglia after facial nerve axotomy (Ladeby et al., 2005). Considering the role of these genes in inflammation, we suggest that those may represent targets to prevent the transition from acute to chronic neuroinflammation. Among them, we highlight DDX3X, since its inhibition leads to a substantial decrease in IL-1β and it is increased on microglia under neurodegenerative conditions and in the AD brain.

DDX3X has been identified as a key regulator for NLRP3 inflammasome in microglial cells by promoting the assembly of ASC and pro-caspase1, leading to inflammasome activation and inflammatory responses (Samir & Kanneganti, 2022; Samir et al., 2019). Beyond, inflammasome activation, DDX3X expression influences microglial functions by regulating the translation of key proteins involved in cell migration and phagocytosis, including STAT1, GNB2 and Rac1 (Guo et al., 2021; Ku et al., 2019; Rastad et al., 2023; Samir & Kanneganti, 2022). This suggests that DDX3X may influence microglial proliferation and their phagocytic activity through its role in inflammasome signaling. Our study provides a strong support that amyloid accumulation in APP/PS1 mice induces a distinct transcriptional and functional program in proliferating microglia governed by NLRP3 inflammasome activation mediated by increased levels of DDX3X. Moreover, we pointed out targets that might serve as new therapeutic approaches not only in AD but also in other CNS disorders, which harbor a chronic inflammatory component, since increased microglia proliferation is a recurrent phenomenon in both acute injuries and chronic neurodegeneration.

## Supplementary Material

Supplementary figure_Supplementary Figure 2.tif

Supplementary figure_Supplementary Figure 4.tif

Supplementary Table 1.xlsx

Supplementary Table 2.xlsx

Supplementary figure_Supplementary Figure 1.tif

Supplementary figure_Supplementary Figure 3.tif

## Data Availability

The datasets generated and/or analyzed during the current study have been made available as Supplementary Information (Supplementary Tables) and as Source Data (GSE173453). Further data are available upon reasonable request from the corresponding author.
