# Supplementary figures and images for: Proliferating Microglia Exhibit Unique Transcriptional and Functional Alterations in Alzheimer’s Disease
Source: ASN Neuro. 2025 May 19;17(1):2506406. doi: 10.1080/17590914.2025.2506406 (PMC12140498; doi:10.1080/17590914.2025.2506406)

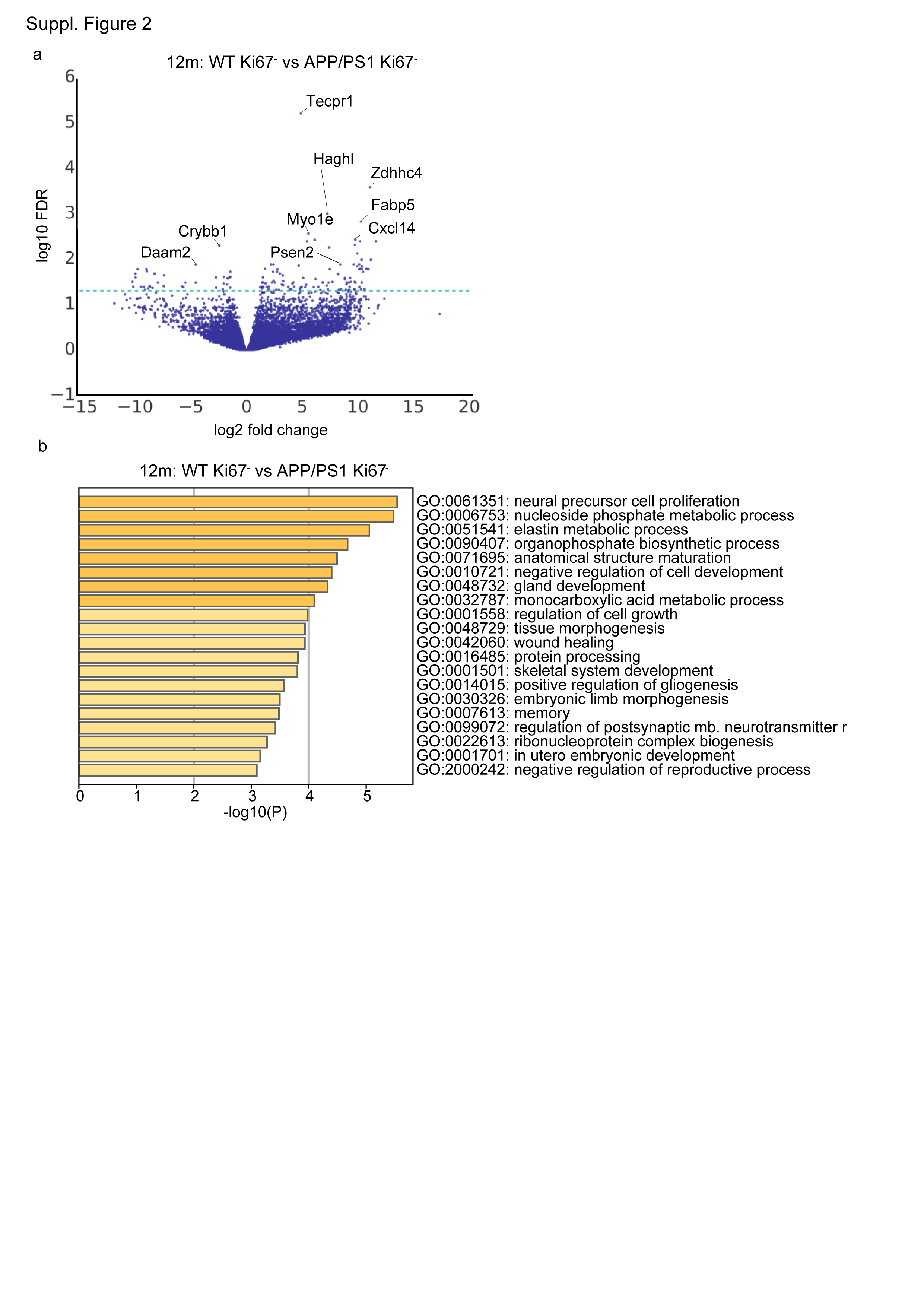

Supplement: Supplementary figure_Supplementary Figure 2.tif [file TASN_A_2506406_SM4212.tif]

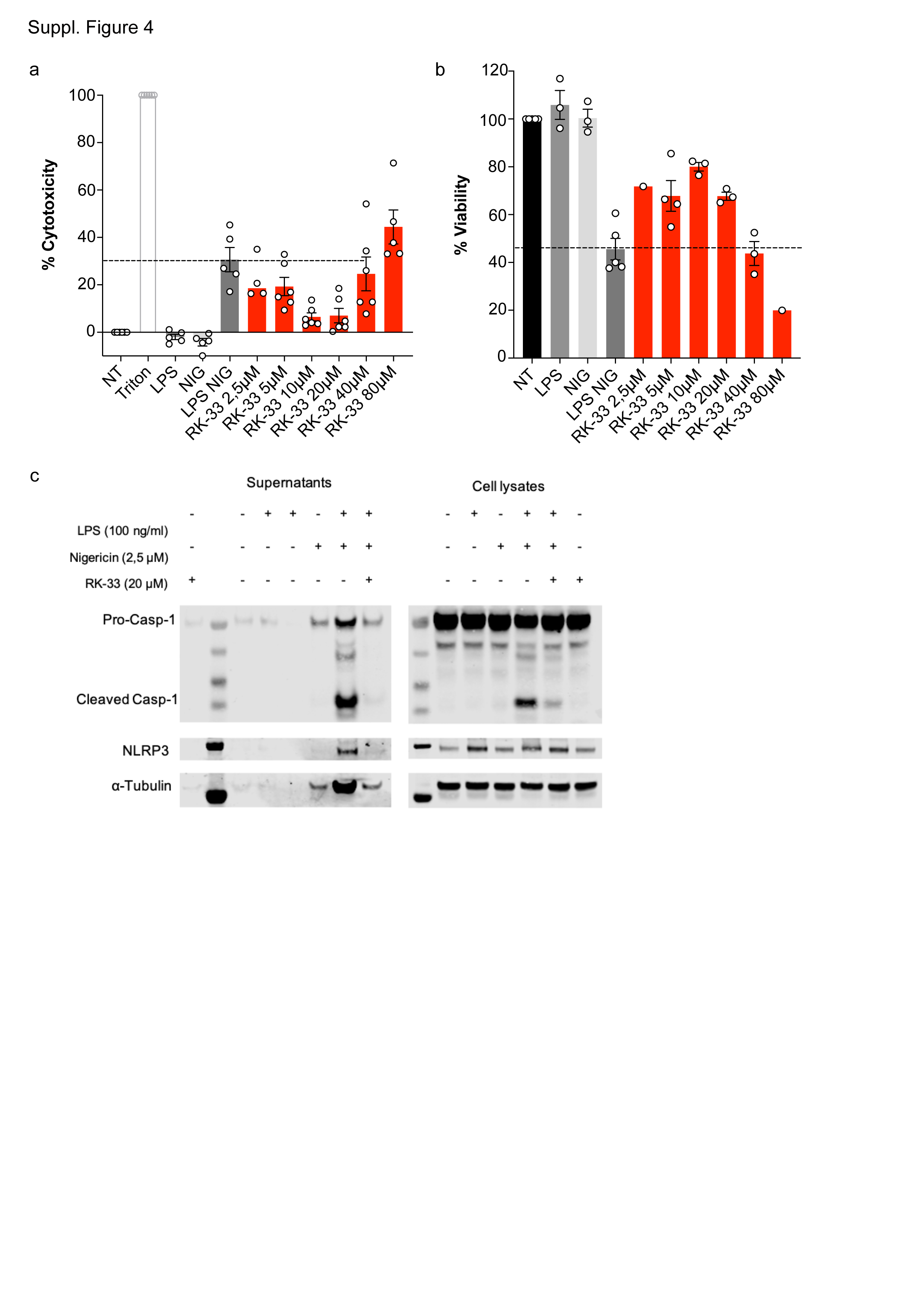

Supplement: Supplementary figure_Supplementary Figure 4.tif [file TASN_A_2506406_SM4211.tif]

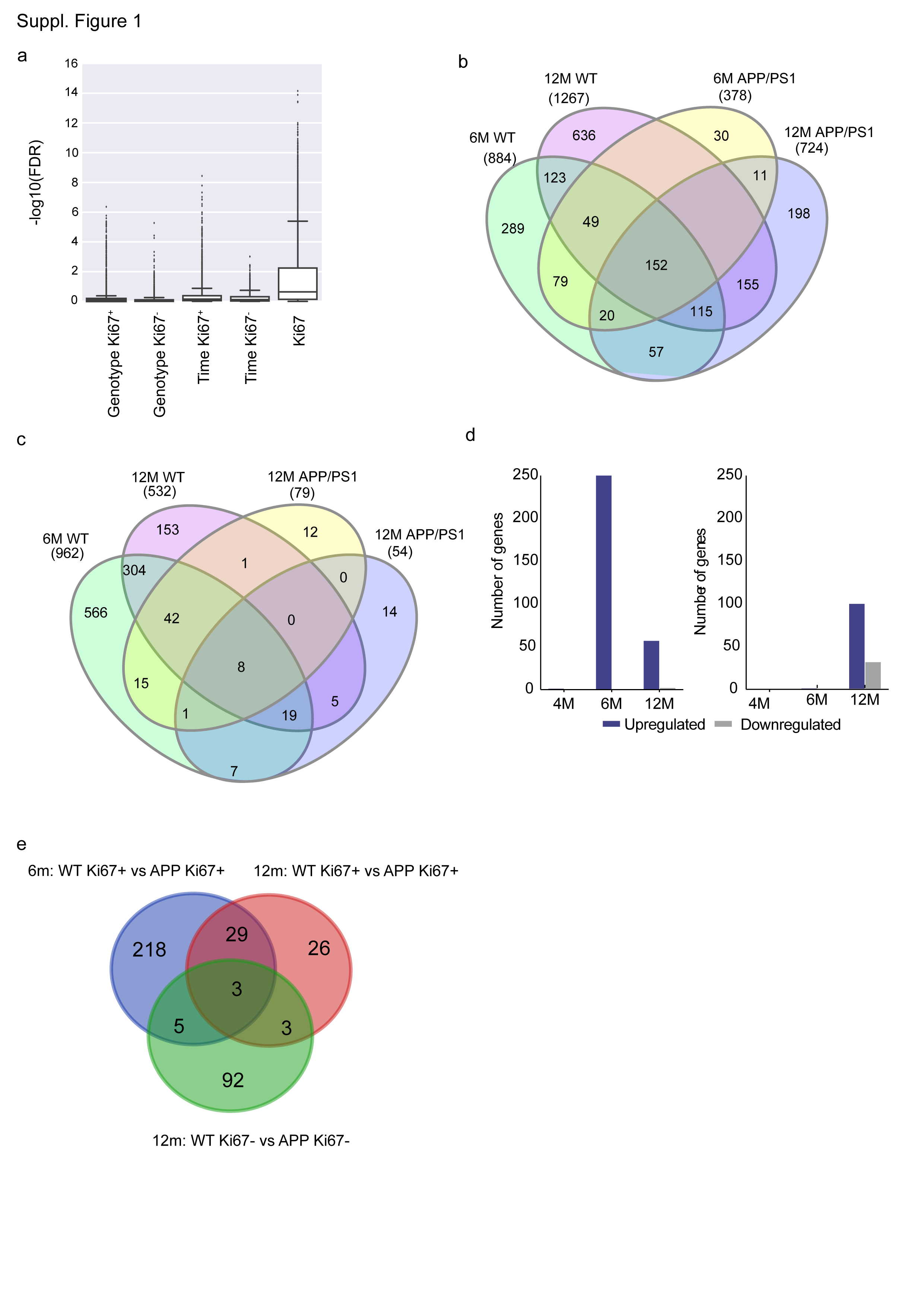

Supplement: Supplementary figure_Supplementary Figure 1.tif [file TASN_A_2506406_SM4208.tif]

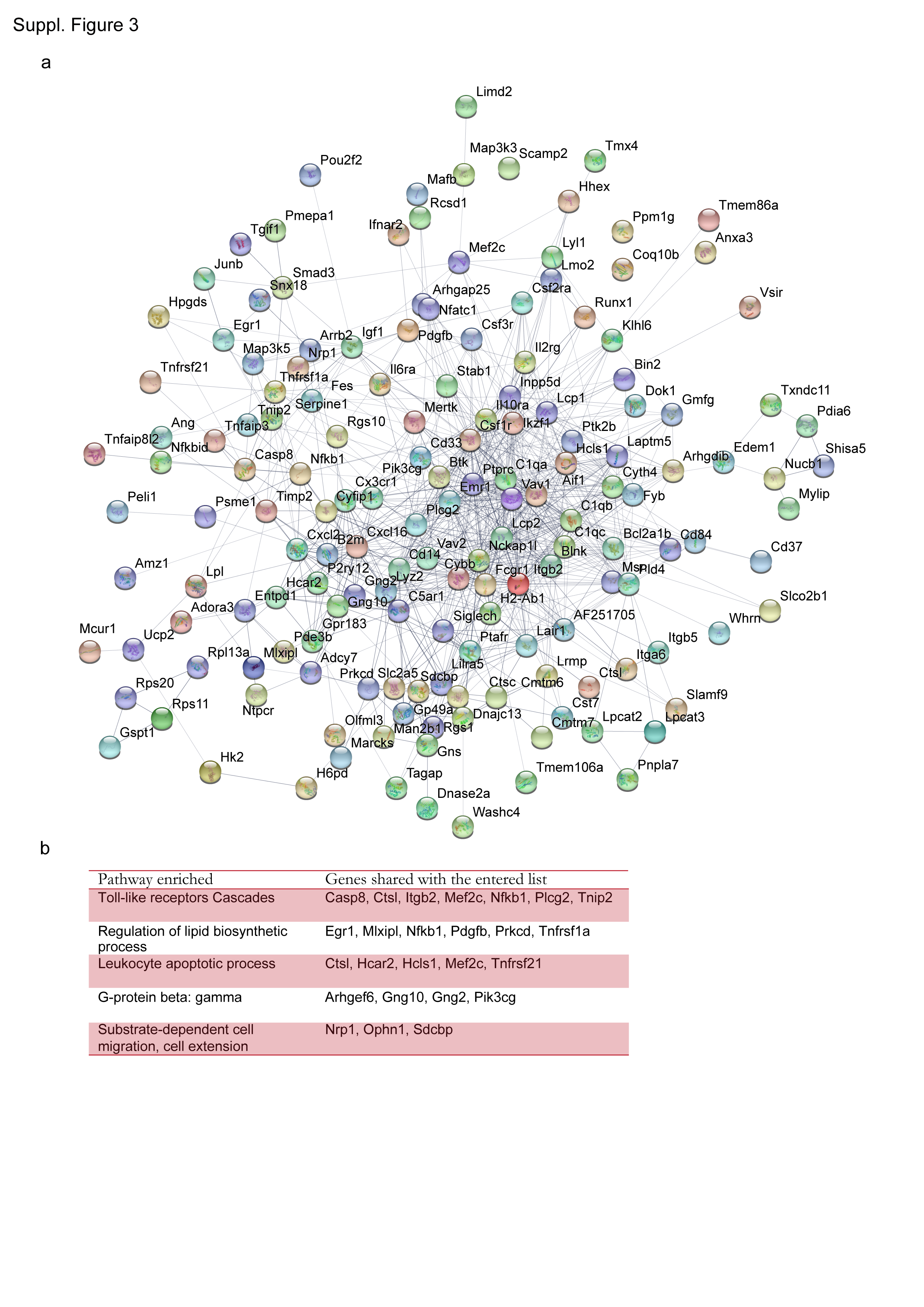

Supplement: Supplementary figure_Supplementary Figure 3.tif [file TASN_A_2506406_SM4207.tif]
